# DUX4 at 25: how it emerged from “junk DNA” to become the cause of facioscapulohumeral muscular dystrophy

**DOI:** 10.1186/s13395-025-00388-0

**Published:** 2025-08-25

**Authors:** Alexandra Belayew, Alberto L. Rosa, Peter S. Zammit

**Affiliations:** 1 Laboratory of Respiratory Physiology, Pathophysiology and Rehabilitation, Research Institute for Health Sciences and Technology, University of Mons, Mons, 7000 Belgium; 2 Laboratorio de Genética y Biología Molecular, Departamento de Farmacología Otto Orsingher, Facultad de Ciencias Químicas, IFEC-CONICET, Universidad Nacional de Córdoba, Córdoba, Argentina; 3 Randall Centre for Cell and Molecular Biophysics, King’s College London, Guy’s Campus, London, SE1 1UL UK

**Keywords:** DUX4, D4Z4, FSHD, Facioscapulohumeral muscular dystrophy, Muscle, Pathology

## Abstract

Double Homeobox 4 (DUX4) is a potent transcription factor encoded by a retrogene mapped in D4Z4 repeated elements on chromosome 4q35. DUX4 has emerged as pivotal in the pathomechanisms of facioscapulohumeral muscular dystrophy (FSHD), a relatively common hereditary muscle wasting condition, although classified as a rare disease. DUX4 contributes to zygote genome activation before its expression is repressed in most somatic tissues through epigenetic mechanisms, including DNA methylation and chromatin modifications. In FSHD, inappropriate activation of *DUX4* expression is driven by a complex interplay of genomic and epigenetic alterations. The ectopic presence of DUX4 in skeletal muscle cells activates genes, viral elements and pathways that are typical of very early embryonic development, disturbing cell function and ultimately contributing to muscle weakness and wasting. This review first traces the history of DUX4, from the FSHD genetic linkage studies in the early 1990s, through to identification and characterization of the *DUX4* gene in 1999. We then discuss the seminal studies that showed how and why *DUX4* is expressed in FSHD and the effects of this ectopic expression in muscle, notably cellular toxicity. Other pathological roles of DUX4, such as participation in cancer and viral infection, are also highlighted. Maintenance of *DUX4* in the genome was explained by discovery of the function of DUX4 in zygotic genome activation to institute the totipotent cells of the embryo. Thus, we encompass the gradual transition of *DUX4* over the past 25 years from being considered a pseudogene in “junk DNA” to becoming central to understanding the molecular pathogenesis of FSHD and the primary focus for FSHD therapeutics.

## Introduction

DUX4 came to prominence during the search for the genetic cause of facioscapulohumeral muscular dystrophy (FSHD). This disorder is the third most common inherited muscular dystrophy after Duchenne muscular dystrophy and Myotonic dystrophy, with an estimated mean prevalence of 5/100,000 and incidence of 0.3/100,000 person-years in Western Europe [1–3]. FSHD is divided into the more common FSHD1 (OMIM 158900) and the rarer (~ 5% of cases) digenic FSHD2 (OMIM 158901). In both cases, pathology is caused by a DUX4 gain of function that requires (1) genetic predisposition: a complete *DUX4* gene with a polyadenylation signal, (2) a particular epigenetic conformation: DNA hypomethylation and ‘open’ chromatin structure allowing *DUX4* transcription. In most somatic cells, *DUX4* is not expressed because it maps in a large array of hypermethylated repeated elements named D4Z4 that is ‘buried’ in compact chromatin. Epigenetic alterations leading to *DUX4* expression result either from a decrease in D4Z4 repeat array size below a critical level (FSHD1) or a less contracted array accompanied by mutation in a gene encoding a protein involved in DNA methylation and/or chromatin modification (FSHD2) [4].

Clinically, FSHD is often described as a descending skeletal muscle weakness and wasting, in most cases with left/right asymmetry [5]. FSHD usually presents in the second decade of life in males but there is evidence of later onset in females [6]. Symptom onset and severity demonstrate inter-patient heterogeneity, which has even been observed in monozygotic twins [7]. Facial weakness is typically the earliest symptom, but weakness in the shoulder girdle and upper arms are the most common presenting symptoms [8–12]. As disease progresses, notable abdominal, lower limb and hip girdle musculature weakness are common [8, 13]. Lower limb weakness can necessitate use of ambulatory aids and wheelchairs [14, 15]. This ‘classical’ FSHD occurs in most patients, with the remainder being atypical, such as a facial sparing variant [11, 16, 17]. Extra-muscular features can include a retinal vascular pathology resembling Coat’s disease [18], and, rarer, sensorineural hearing loss [19] and asymptomatic electrocardiogram abnormalities [20]. Infantile onset (< 10 years of age) accounts for ~ 10% of cases, usually with rapid ‘classical’ progression [21] and higher prevalence of extra-muscular features [22].

Newcomers to the field of FSHD often think that DUX4 history started in 2010 with the key publication of a large multicentre genetic study led by Silvère van der Maarel [23], showing that a permissive allele (4qA) provides a polyadenylation signal (PAS) to stabilize *DUX4* mRNA allowing for translation to the muscle-toxic DUX4 protein [23]. This “unifying theory of FSHD”, was built upon a body of research conducted over many years, which was instrumental to its formulation. Here, we first outline the research that shed light on many aspects of the genetics and molecular biology of FSHD, which contributed to the current model of pathogenesis. We describe the history of DUX4, starting with the FSHD genetic linkage studies of the early 1990s, and continuing through three key milestones: identification of the *DUX4* gene in the D4Z4 repeat array in 1999 [24], discovery of DUX4 toxicity [25], *DUX4* mRNA characterization with a PAS 3’ of D4Z4 and DUX4 protein detection in FSHD muscle cells [26]. How our understanding of DUX4 has gradually shifted from being considered a pseudogene in “junk DNA”, to acceptance as fundamental to molecular pathology in FSHD is then discussed [4, 27–29]. We finish by highlighting some of the outstanding questions about DUX4 and its role in FSHD.

## The foundations of FSHD clinical symptoms and inheritance

In the mid-1800s, neurologists believed that muscle paralysis or atrophy could only result from a nerve lesion. The first account of a primary muscle disease was probably by Edward Meryon in 1852, who described 8 boys from 3 families with early onset muscle wasting. This seminal study reported muscle wasting without apparent involvement of nerves, which was both inherited and X-linked, and was likely Duchenne muscular dystrophy [30]. At around this time, Jean Cruveilhier described the autopsy of an 18-year old man who presented a severe facioscapulohumeral muscle wasting syndrome with unaffected brain, spinal cord or peripheral nerves [31], now considered the first case of FSHD [5]. Later, when Guillaume-Benjamin-Amand Duchenne (de Boulogne) published his ground breaking work on muscular dystrophies, he also included the classification of “l’atrophie musculaire graisseuse progressive de l’enfance” [32] that included facial muscle weakness and a descending progression of muscular involvement: essentially highlighting the muscular features of FSHD. A few years later, Louis Théophile Joseph Landouzy and Joseph Jules Dejerine described patients with selective facial, then shoulder/upper arm, followed by trunk/pelvic musculature involvement [33, 34]. Muscle atrophy with light sclerosis and adiposity was noted from post-mortem examination of a 24-year-old patient. Importantly, brain, spinal cord, peripheral nerve and intramuscular nerve endings were normal, indicating no neurological ‘*disturbance*’ [35]. This pedigree, and other cases, led Landouzy and Dejerine to term the disorder ‘*facioscapulohumeral type of progressive myopathy*’, extend the definition to include infant onset cases and encompass both facial muscle and/or shoulder girdle weakness [33, 34]. This explains why FSHD is also known as Landouzy-Dejerine muscular dystrophy. Wilhelm Erb independently confirmed that the muscle wasting conditions that he called ‘dystrophia muscularis progressiva’ were primarily muscle disorders and so distinct from secondary progressive muscular atrophy due to spinal cord disease [36, 37]. Based on initial muscle involvement, muscles affected and clinical symptoms, Erb classified four categories of “dystrophia muscularis progressiva” that included the facioscapulohumeral type described by Landouzy and Dejerine [38].

Landouzy and Dejerine also found that FSHD was inherited in a five-generation pedigree with the proband’s father, younger brother and sister similarly affected. Typical Mendelian inheritance with complete penetrance and highly variable expression was described in the 1950s by Frank Tyler and Fayette Stephens with a study of 1249 descendants of a man who emigrated to Utah from England in the 1850s [8]. It is of note that the Tyler and Stephens work was funded by the first NIH grant, after the Public Health Service Act of 1944 allowed the NIH to give grants to researchers [39]. In this Utah kindred, FSHD is linked to a 20-kb D4Z4 repeat array in 4q35, conserved in multiple, distantly-related branches, confirming the meiotic stability of the deletion [40]. George Padberg further explored the Mendelian inheritance and was central to the genetic search for the FSHD locus. During his PhD thesis, he toured The Netherlands to document families comprising affected and non-affected individuals and obtained numerous blood samples correlated with clinical description and family history [5].

## The hunt for the genetic locus linked to FSHD

**Fig. 1:**
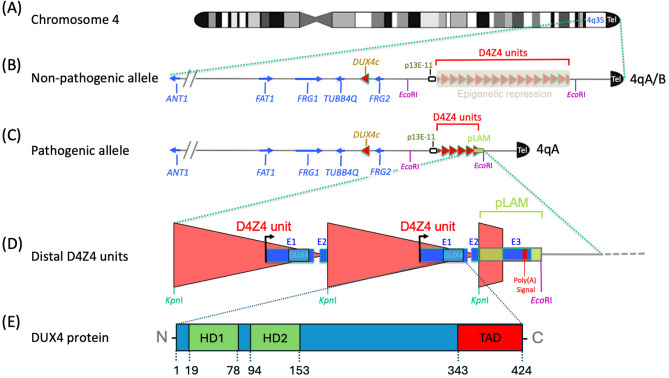
The FSHD locus at 4q35 contains a tandem D4Z4 unit array, each with a DUX4 ORF. (**A**) The FSHD locus is located adjacent to a telomere (Tel) of chromosome 4 at 4q35. (**B**) The locus usually contains a microsatellite array of > 11 D4Z4 units (red triangles) arranged head to tail, associated with epigenetic repression. This can be isolated on an *Eco*RI fragment identified by hybridization to the p13E-11 probe. Sequence differences telomeric to the *D4Z4* repeat array define either a 4qA or 4qB haplotype. Centromeric to the locus are neighbouring genes including *FRG2*,* DUX4c*,* TUBB4q*,* FRG1*,* FAT1* and *ANT1.* (**C**) In FSHD1, there is a reduced number of D4Z4 repeats to between 1 − 10 units, which leads to epigenetic derepression. FSHD1 also requires a 4qA haplotype in *cis* with the contracted array, containing the pLAM region (green box). Shortening of the D4Z4 repeat array generates a smaller *Eco*RI fragment identified using the p13E-11 probe. (**D**) A complete 3.3-kb D4Z4 unit is delimited by *Kpn*I sites. Each D4Z4 unit contains the promoter, transcription start site, and entire open reading frame for the *DUX4* retrotransposed gene in exon 1 (E1), together with the non-coding exon 2 (E2). The pLAM region on the 4qA haplotype provides intron 2 and exon 3 (E3) containing a polyadenylation (Poly(A)) signal required to stabilise *DUX4* mRNA, allowing for its translation. (**E**) The main regions of DUX4 protein include the two DNA binding homeodomains (HD1 and HD2) and the transactivation domain (TAD) at the carboxyl-terminus. Locations of domains are given with respect to amino acid residues

Shortly after the discovery that mutations in the *DMD* (*dystrophin*) gene caused Duchenne muscular dystrophy [41], an international consortium was established to find the ‘FSHD’ gene [42]. This initially led to exclusion of > 80% of the genome [43]. Advent of multiallelic microsatellite markers facilitated more efficient screening and one of these (*Mfd22*) displayed linkage at 13 centi morgan (cM) from the FSHD locus, with a LOD score above 6. This was the first genetic linkage success with such microsatellite markers. The corresponding locus D4S171, was assigned to chromosome 4 by the Peter Harper and Padberg labs in 1990 [44, 45]. Mapping was refined when cosmid 13E, comprising genomic DNA from that region, was isolated in a collaboration between the groups of Robert Williamson, Padberg and Rune Frants [46]. Different repeated sequences in this region strongly complicated the search. However, extensive subcloning finally isolated an almost single copy probe termed p13E-11 (D4F104S1, formerly D4S810), that recognised *Eco*RI restriction fragments (Fig. 1) of up to ~ 40 kb on Southern blot [46]. Association was confirmed by examining DNA from eight sporadic FSHD patients who had D4F104S1 *Eco*RI fragments that were shorter than 30 kb, while their unaffected parents had much longer fragments [46] and in a mosaic individual who passed on the rearranged D4F104S1 fragment to his affected son [47]. These seminal contributions from Frants’ group were key to the diagnosis of FSHD, anticipating that the p13E-11 probe “*has immediate diagnostic value*” [46]. The authors were also optimistic about identifying the gene responsible for FSHD, stating in 1992 that “*the cloning of the FSHD gene should now be imminent*” [46].

Importantly, 13E was isolated by a screen in search for homeobox genes by hybridization to a homeobox probe [46], and each D4Z4 unit was bound [48]. Fine restriction mapping of the *Eco*RI fragment detected with p13E-11 hybridization by the Frants and Jane Hewitt groups revealed that it contained multiple copies of this homeobox-containing element. Digestion with *Kpn*I delineated these as 3.2-kb repeat units, which were then termed D4Z4 [48–50]. Crucially, the proximal and distal sequences were identical on D4F104S1 *Eco*RI fragments from both unaffected and FSHD-affected individuals [50]. This *Eco*RI fragment, however, was shorter in FSHD and differed in size by multiples of 3.2-kb, suggesting rearrangements by homologous recombination resulting in loss of entire D4Z4 units [50]. Thus, the restriction map of this genomic fragment started with the 5’ *Eco*RI site, followed by the D4F104S1 single copy sequence detected by p13E-11, then by several homeobox-containing 3.2-kb D4Z4 units delineated by *Kpn*I sites, and ended with the 3’ *Eco*RI site (Fig. 1). The region between the distal *Kpn*I and *Eco*RI sites had been identified by a probe called pLAM1 and was either 1.5 or 2.9 kb long [50]. This telomeric region became known as pLAM, beginning with the *Kpn*1 site of a truncated D4Z4 unit, and containing a distal part composed of 68-bp tandem repeats similar to the *Sau3A* repeat family [50] (Fig. 1). The D4Z4 repeat array also begins with an incomplete D4Z4 unit (911-bp) located 5’ of the first *Kpn*I site [51].

## D4Z4 sequence reveals an open reading frame encoding two homeodomains

The full sequence of *D4Z4* was published by a joint effort of the Hewitt, Frants and Williams labs in 1994 [52], following a partial sequence published earlier in that year [53]. A *D4Z4* unit was 3,261 bp long, hence the revision in unit size to 3.3-kb. The sequence was GC-rich (71% G + C), containing GC-rich repeats designated *LSau* and a low copy repeat termed *hhspm3*, followed by two homeoboxes presenting 67% sequence identity between them and separated by 45-bp [52]. The encoded proximal homeodomain was named HD1 and the distal, HD2 (Fig. 1), sharing 52% amino acid sequence identity [52]. These two homeodomains had the greatest similarity to homeodomains of the paired and orthodenticle classes [52]. In searching for open reading frames (ORF), while none went through an entire D4Z4 unit, a long ORF encoding both homeodomains was detected. However, no evidence for expression was found from screening cDNA libraries [52]. Interestingly, the homeoboxes from *D4Z4* were identical in sequence (across 405 bp) to the centromeric, inverted homeobox-containing region [52] previously identified with the homeobox probe [48]: this was later found to be part of the *DUX4c* gene.

## Homologous D4Z4 tandem repeat arrays are on both chromosomes 4 and 10

Location of the tandem array of D4Z4 repeats on chromosome 4 was further refined to heterochromatin adjacent (within 215 kb) to the 4q telomere at 4q35 [53, 54]. The p13E-11 probe hybridized to two nonallelic *Eco*RI polymorphic fragments, generating a total of four fragments, of which only two were assigned to 4q35. The mystery was solved by the Frants and Luciano Felicetti groups who showed that the other two *Eco*RI fragments delimited tandem D4Z4 units located instead on chromosome 10q26 [55, 56]. These 4q35 and 10q26 homologous subtelomeric regions arose from duplication events and evolved independently [55, 57, 58]. Importantly, the D4Z4 repeat array at 10q26 was not associated with FSHD [56] although D4Z4 units on chromosomes 4 and 10 had 98–100% sequence identity. D4Z4 units on chromosome 10 had a *Bln*I restriction site absent in the repeats on chromosome 4 [59], which contained a unique *Xap*I site. This polymorphism permitted detection of 4/10 inter-chromosomal exchanges and cases of hybrid chromosome 4 and 10-derived D4Z4 units [60]. A puzzle at the time however, was that this region had all the characteristics of so called non-functional “junk DNA” [61].

## FSHD is only associated with contracted D4Z4 arrays on chromosome 4

The four bands observed on Southern blot when human genomic DNA was digested with *Eco*RI and hybridized with p13E-11 were thus explained: two originating from chromosome 4 and two from chromosome 10. Conversion of *Eco*RI fragment size (kb) to D4Z4 unit number is determined by subtracting 8.5 kb (the combined size of the 6 and 2.5 kb DNA segments flanking the D4Z4 array) and dividing by 3.3, the D4Z4 unit size in kb [62]. Most *Eco*RI fragments were 38 kb to > 300 kb in length, so accounting for ~10 to > 100 D4Z4 units. FSHD however, was associated with at least one D4F104S1 *Eco*RI fragment of 10 to 38 kb, so containing 1 − 10 D4Z4 units, as shown by Frants and Gert-Jan van Ommen's groups [50, 63].

Association was then found between D4F104S1 *Eco*RI fragment size and age of disease onset, with smaller fragments in the range of 10–18 kb (1–3 D4Z4 units) being severe childhood cases, 18–34 kb (3–8 units) usually associated with typical teenage onset, while ~ 30–38 kb (7–10 units) often associated with late onset [64, 65]. Also, high inter- and intra-familial variability in clinical presentation was noted, which even occurred in monozygotic twins [7, 66, 67]. Significantly, Rossella Tupler and colleagues found a healthy individual with a total loss of D4Z4 units on one 4q35 allele, indicating that FSHD was not associated with haploinsufficiency of a key gene(s) at the 4q subtelomeric region [68]. In fact, this enigmatic result underpinned the cryptic pathogenic mechanism of the disease, as it indicated either a dominant-negative or gain-of-function effect.

FSHD was only associated with a contracted D4F104S1 *Eco*RI fragment on chromosome 4 [69] on which the D4Z4 repeat array was located in heterochromatin and adjacent to the 4q telomere [53, 54]. Because homologous D4Z4 repeat arrays were found on both chromosomes 4 and 10 but only chromosome 4 was linked to the pathology, the mysterious FSHD-causing gene(s) was proposed to map outside of the D4Z4 repeat array and its expression to be subject to a position effect [52]. In this model, expression levels of a gene result from the degree of telomeric heterochromatin extension onto it, as initially described in yeast [70, 71]. Telomeres are composed of a 6-bp repeated sequence (TTAGGG in human) associated with heterochromatin, and repeat copy number is correlated with extension of heterochromatin outside of the telomeric region. Changes in the D4Z4 repeat array length/chromatin structure was proposed to favour or block telomere heterochromatin extension to the unknown FSHD gene(s) and so affect its expression [52]. A decade later, this model proved correct for *DUX4*, where telomere length alterations in isogenic FSHD myoblasts/tubes showed increased *DUX4* gene expression inversely proportional to telomere length [72].

## FSHD is only associated with the 4qA haplotype distal to the contracted D4Z4 array

Another important piece of the puzzle was contributed by Van der Maarel’s group, who observed that 4q35 subtelomeric DNA sequences could be segregated into 4qA and 4qB haplotypes, found with approximately equal frequency in the general population [73]. In 4qA, the telomeric-most complete 3.3-kb D4Z4 unit is followed by pLAM, which starts with the truncated D4Z4 unit and ends with only the first portion of an 8 kb of 68-bp beta-satellite repeated DNA, which itself is followed by a 1-kb divergent (TTAGGG)_n_ array (Fig. 1). In the 4qB allele by contrast, the D4Z4 array ends with just the first 570 bp of a truncated D4Z4 unit but no satellite repeats [73]. The D4Z4 array on 10q was also followed by a 68-bp beta-satellite array, with 4qA and 10qter subtelomeres being > 98% identical, so closer in sequence than the 92% similarity between 4qA and 4qB [74]. Importantly, FSHD was only associated with contracted D4Z4 tandem arrays in *cis* on a 4qA haplotype [73] (Fig. 1).

## The D4Z4 array is normally epigenetically repressed

The D4Z4 array contained CpG islands [48] and was located within heterochromatin adjacent to the 4q telomere [53, 54]. Sequencing had shown that each D4Z4 unit was GC-rich (71% G + C) and contained a CpG/GpC dinucleotide ratio of 0.8 and GC-rich *hhspm3* [52] and *LSau* repetitive sequences [52, 53], associated with heterochromatin. D4Z4 was noted to bind a multiprotein complex consisting of YY1, together with HMGB2 and nucleolin, that suppressed expression of a linked reporter gene [75]. Furthermore, van der Maarel and colleagues reported that while many CpG dinucleotides in the D4Z4 array were methylated in unaffected individuals, there was reduced DNA methylation in contracted alleles, associated with an epigenetic state more conducive to gene expression [76, 77]. Intriguingly, this was also the case in FSHD2, where larger “non-contracted” D4Z4 arrays on both chromosomes 4 and 10 were hypomethylated [78]. Later, a reduction in binding of HP1γ and cohesin at D4Z4, accompanied by loss of the histone mark H3K9 trimethylation, was found to hallmark FSHD1 [79]. Davide Gabellini’s lab revealed that contraction of the D4Z4 array resulted in loss of Polycomb silencing and gain of Trithorax activation. This led to expression of a long non-coding RNA (lncRNA) termed *DBE-T* that could recruit Ash1L to facilitate gene derepression [80]. Thus, in FSHD, the D4Z4 array exhibits a less repressed epigenetic state, and the *DUX4* gene can be stochastically activated in rare myoblasts or myonuclei by ‘available’ regulatory elements (e.g. DUX4 myogenic enhancer 1 and 2) [81] and transcription factors such as Sp1, p53, WDR5 and SIX family members [24, 82–84]. Indeed, Peter Jones and colleagues showed that the epigenetic status assessed via the DNA methylation of the distal D4Z4 unit on a 4qA haplotype correlates with disease: hypomethylation in FSHD1 but intermediate methylation in non-manifesting, and hypermethylation in healthy, individuals [85]. Moreover, the even lower methylation in FSHD2 allowed the Jones lab to develop a differential diagnosis method based on the presence of a 4qA allele, and the distal D4Z4 methylation level [86].

## Discovery of the *DUX4* gene fails to set the field alight

So, what made the combination of a small D4Z4 array and a 4qA haplotype pathogenic? Nine years elapsed from mapping the chromosomal location of the FSHD locus to chromosome 4 [44, 45], to discovery of the *DUX4* gene by Alexandra Belayew’s group [24, 87] based on the Hewitt publication of the D4F104S1 *Eco*RI fragment sequence [52]. A large ORF containing the two homeoboxes was within each 3.3-kb D4Z4 unit but neither a promoter nor a cDNA clone with sequence identical to the D4Z4 ORF was found [51, 52]. Prophetically, it had been suggested in Hewitt et al. that “*the overall structure of D4Z4 makes it unlikely to encode a functional protein; however*,* it cannot be ruled out that one copy of the repeat may produce a protein*” [52].

Searching for target genes of Helicase-like Transcription Factor (HLTF/SMARCA3) via chromatin immunoprecipitation identified a 182-bp fragment containing a putative TATAA box that was named *HLTF target 1* (*HEFT1*) [87]. Multiple potential *HEFT1* promoter regions were found in 3.3-kb elements repeated throughout the genome in so called “junk DNA” including at D4Z4. This *HEFT1* promoter had 87% sequence identity with a region inside the D4Z4 ORF, upstream of the two homeoboxes. The *HEFT1* TATAA box corresponded to a functional variant TACAA sequence in D4Z4, generating a shorter ORF with a potential start codon 135 bp downstream from that of Hewitt et al. 1994 [52] but still in frame with the two homeoboxes. The ORF sequence known at the time encoded a putative 424-residue protein with two homeodomains [87]. Both promoter and ORF were also present in a 17.5 kb fragment of patient genomic DNA provided by the Frants group [24]. Of note, several GC quadruplex structures (G4s) have been identified in D4Z4 units and *DUX4* promoter/enhancer regions [88, 89] and recent studies demonstrate that HLTF interacts with and destabilizes such G4s to facilitate error-free DNA replication [90].

This putative gene within each D4Z4 unit was named *“Double Homeobox on chromosome 4”* or *DUX4* [24] (Fig. 1). The *DUX4* promoter was active in human rhabdomyosarcoma cells and depended on the TACAA and a GC box [24]. Intriguingly, this promoter overlapped with the *hhspm3* sequence identified in 1987 by Melanie Ehrlich [91] as hypo-methylated in sperm cells, an epigenetic feature that was shown 23 years later to associate with *DUX4* expression in testis [92]. At the time of discovery however, many considered *DUX4* a pseudogene due to the mutated TATAA box, lack of both introns and PAS, and, above all, inability to identify either a cDNA corresponding to its mRNA or an encoded protein. Attempts at amplifying *DUX4* mRNA by RT-PCR picked up highly similar RNAs with no, or minimal, ORFs derived from hundreds of homologous 3.3-kb repeats dispersed throughout the genome, mostly on all acrocentric chromosomes, interspersed with ribosomal RNA gene clusters [53, 87, 93–95]. This reinforced the notion that the 4q35 D4Z4 array was devoid of a gene.

## Position effect variegation and effects on potential candidate FSHD genes

The hypothesis was advanced that D4Z4 was instead involved in position effect variegation, with changes in its chromatin structure affecting expression of neighbouring genes [75, 96]. Position effect variegation was discovered in *drosophila* using an eye colour phenotype. When the *white* gene was inserted in the vicinity of heterochromatin, this inhibitory conformation could spread to different extents in each cell, resulting in a variable pattern of *white* gene expression causing mosaic red eye pigmentation [97].

Only a few genes have been mapped in the vicinity of the FSHD locus: the gene encoding mitochondrial channel Adenine Nucleotide Translocator (*ANT1*, also known as *SLC24A4)* in 1989 [98, 99], then *FSHD Region Gene 1* (*FRG1*) in 1996 [100], *TUBB4Q* in 2000 [101] and *FRG2* in 2002 [74] (Fig. 1). The Tupler group reported that the closer a gene was to the D4Z4 units, the more it was inhibited in healthy control muscle and inappropriately overexpressed in FSHD [75]. According to this study, each D4Z4 element harboured a repressor named DBE, and DBE multimerization inhibited a linked reporter gene. Consistent with this, 4q35 sequences were shown to be hypomethylated, and so epigenetically derepressed, on chromosome 4 variants associated with FSHD [76]. However early transcriptomic profiling using microarrays did not find misregulation of these genes in FSHD muscle [102]. A homologous *DUX4c* (centromeric, also known as *DUX4L9*) gene had also been mapped to a single truncated, inverted D4Z4 unit proximal to *FRG2* [48, 103]. The encoded 46-kDa protein had high sequence identity to DUX4 but with a shorter carboxyl-terminal region of 32 residues (instead of the 82 residues of DUX4) due to a frameshift in the ORF that made DUX4c a significantly less potent transcriptional activator than DUX4 [52, 103–105]. *FAT Atypical Cadherin 1* (*FAT1)* is also near the FSHD locus and because loss-of-function mutations were found in patients who had D4Z4 copy numbers close to the normal range, they were suggested to cause FSHD, despite the disease dominant transmission [106] (Fig. 1).

## DUX4: the wilderness years

From its discovery in 1999 [24], there followed a period until 2010 when *DUX4* was not widely considered relevant to FSHD, except by a few hardy acolytes. Indeed, the next publication to include the word DUX4 in the abstract was 4 years later in 2003 [107], while DUX4 did not make it into a title until 2005, debuting in a published meeting abstract [108] and then a peer-reviewed paper in 2006 [109].

*DUX* genes from 3.3-kb repeat elements located on other chromosomes were actively transcribed and *DUX1* generated a protein with DNA binding activity [87, 93]. The *DUX4* promoter activated a linked luciferase reporter gene (*DUX4-luc*) in human rhabdomyosarcoma and C2C12 myoblasts, activity that was strongly reduced by mutations in either its TACAA box or a 5’ GC box binding Sp1 [24]. However, the DBE repressor [75] overlapped this *DUX4* promoter. DBE bound a protein complex including HMGB2 and Nucleolin associated with the transcription factor YY1 that can act as either an inhibitor or activator. In HeLa cells, a mutation that suppressed YY1 binding and so repression by DBE, activated a linked reporter gene [75]. Confusingly, this same mutation had no impact on *DUX4-luc* activity in murine C2C12 myoblasts, indicating that DBE was not a repressor in muscle [110]. Furthermore, the encoded DUX4 protein had a carboxyl-terminal domain with powerful transcriptional activity in the yeast one hybrid system and was localised to the nucleus when expressed in C2C12 myoblasts [24, 110]. Crucially, an antiserum raised against a DUX double homeodomain detected several spots on immunoblotted 2D gels, one of which was in primary myoblast extracts from an FSHD patient but not from unaffected individuals [110]. The *DUX4* ORF was later found to be evolutionarily conserved by the Hewitt group, prompting the statement in 2007 that “*Together with the conservation of the DUX4 ORF for > 100 million years*,* this strongly supports a coding function for D4Z4 and necessitates re-examination of current models of the FSHD disease mechanism*” [111]. Indeed, transfecting C2C12 myoblasts with D4Z4 units had been shown to perturb myogenic differentiation, although *DUX4* mRNA could not be detected via RT-PCR [107].

Another important piece of the puzzle came from the cancer field in 2006. A novel chromosomal translocation t(4;19)(q35;q13) in Ewing-like sarcomas generates a hybrid oncogene containing most of the *CIC* gene fused in frame to the 3’ region of the *DUX4* ORF at 4q35 [109]. The encoded CIC–DUX4 chimeric protein retained the CIC DNA-binding domain and its target genes. Interestingly, while CIC-DUX4 was a better transcriptional activator than wild type CIC, the DUX4 carboxyl-terminal domain alone was even more effective [109]. This key study also implied that there may be a functional PAS somewhere downstream of *DUX4* at 4q35 [109].

## DUX4 expression is cytotoxic

**Fig. 2:**
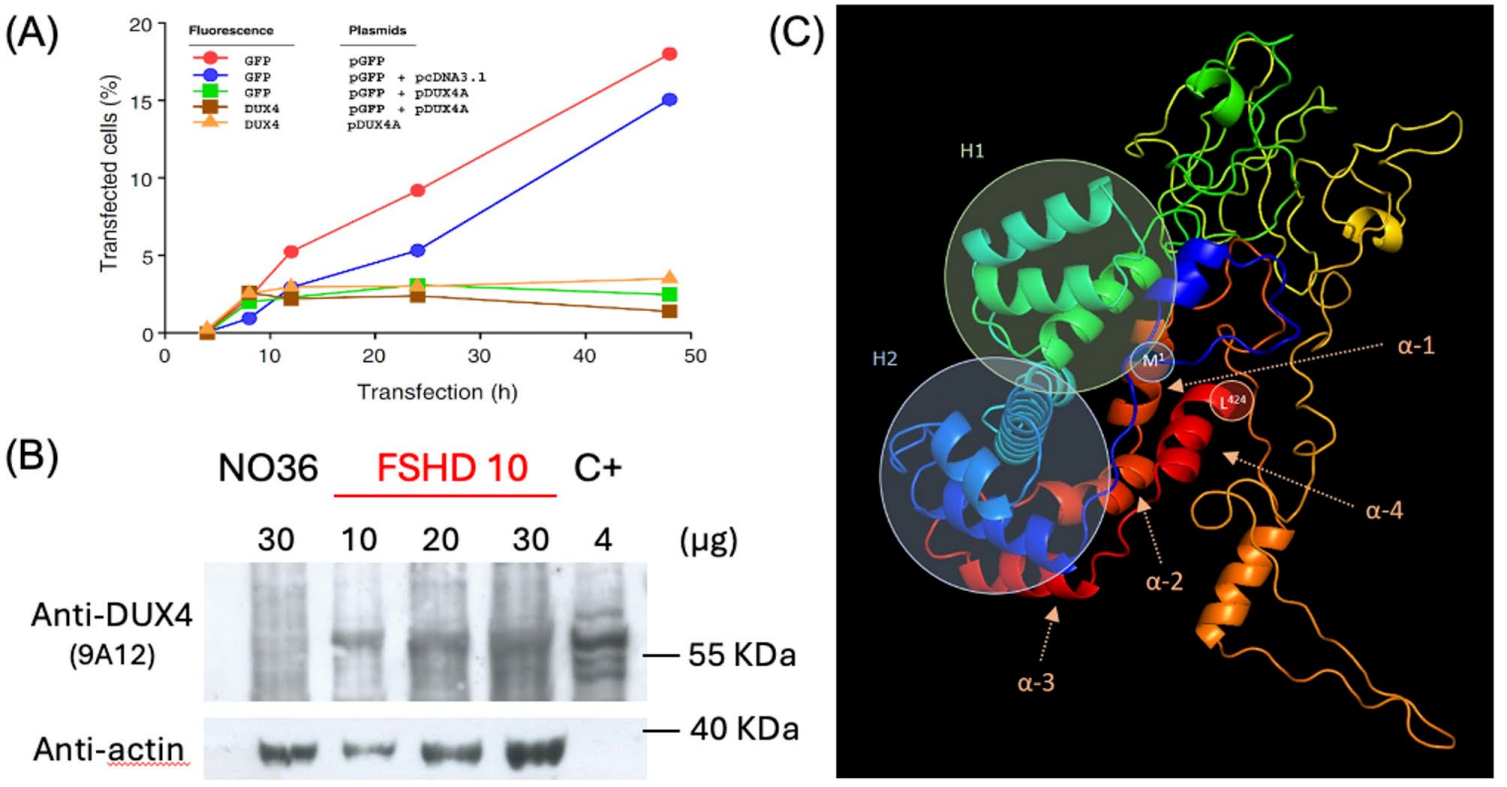
Milestones in the journey to DUX4 detection and function (**A**) DUX4 toxicity as shown in co-transfection experiments. Hep2 cells transfected with a GFP reporter gene (*pGFP*) with or without empty control plasmid (*pcDNA3.1*) present increasing percentage of cells with GFP fluorescence over 50 h. In contrast, the proportion of GFP fluorescent cells after co-transfection with *pGFP* and a *DUX4* expression plasmid (*pDUX4 A*) reached a plateau after 8 h, corresponding to the time cytotoxic DUX4 protein could be detected by immunofluorescence, as well as in cells transfected with *pDUX4 A* alone. From a poster presented by the Rosa group at the FSHD-IRC in Los Angeles in 2003 and published in Kowaljow et al. 2007 [25] (**B**) First immunodetection of DUX4 protein with MAb 9A12 on a Western blot of proteins extracted from FSHD (FSHD10) but not healthy (NO36) myoblasts, with a positive control (C+) of TE671 cells transfected with a *pCI-Neo-DUX4* expression vector (loaded protein amounts are indicated). Actin immunodetected with a rabbit polyclonal was used as a loading control. Performed by Alexandra Tassin for Supplemental Fig. 7 of Dixit et al.. 2007 [26] (Copyright (2007) National Academy of Sciences, U.S.A.) (**C**) The secondary structure of DUX4 using the trRosetta algorithm and PyMOL software (https://pymol.org/2/) reveals an intrinsically disordered protein with three α-helical domains at each homeodomain (H1 and H2) and four α-helical domains in the carboxyl-terminal region (α-1, α-2, α-3, and α-4). The positions of the amino-terminal methionine (M1) and carboxyl-terminal leucine (L424) are indicated. Generated by Alberto L. Rosa for Supplemental Fig. 1 of Quintero et al. 2022 [158]

A breakthrough was demonstration that DUX4 was cytotoxic by Alberto L. Rosa’s group, with a clear dose-response (Fig. 2A) [25]. Rabbit sera raised against synthetic DUX4 short peptides revealed its nuclear localization in transfected cells in vitro. These *DUX4*-expressing cells showed apoptotic features such as Annexin V staining, caspase 3 activation and emerin redistribution at the nuclear envelope [25]. Ubiquitous *DUX4* expression in vivo was also found incompatible with normal Drosophila, zebrafish and Xenopus development, confirming that DUX4 was cytotoxic across species [112–114]. Such observations indicated that the *DUX4* ORF was linked to FSHD pathogenesis [27] and suggested that the pathogenic mechanism was different from the prevailing position variegation effect model [75].

Why would high D4Z4 copy number not give rise to more *DUX4* expression and so cause FSHD? A clue came from early attempts at producing “giant” transgenic mice with additional growth hormone gene copies [115] that revealed integration of high copy number concatemers was associated with less growth (although genome integration site effects could not be discounted). Similarly, mice generated with different copy numbers of a *lacZ* reporter gene at the same chromosome location only exhibited β-galactosidase activity if they carried small *lacZ* copy numbers: these were associated with low DNA methylation and decreased chromatin compaction [116]. This led Belayew and Hewitt to hypothesize that compact chromatin developed on large D4Z4 repeat arrays, preventing *DUX4* gene expression and FSHD development, while short D4Z4 arrays favoured chromatin opening and *DUX4* transcription [24].

## Initial characterization of *DUX4* mRNAs from D4Z4

A strategy to specifically detect *DUX4* mRNA by RT-PCR, despite the hundreds of highly similar *DUX* genes/3.3-kb elements transcribed in the human genome was developed by the Belayew/Coppée group. Transfected murine C2C12 myoblasts with human genomic plasmids containing 1 or 2 D4Z4 units revealed 1.4 and 1.5 kb mRNAs on Northern blot when hybridized to a double homeobox probe, so demonstrating expression from the native *DUX4* promoter. Two transcription start sites were found by rapid amplification of cDNA ends (5’RACE). 70% mapped to an initiator 3’ of the TACAA box and 5’ of the ATG start codon while 30% intriguingly mapped 90 bp upstream, in the DBE repressor element [75] further indicating its inactivity in myogenic cells. Multiple 3’ ends existed downstream from the STOP codon. mRNAs with the full *DUX4* ORF could be amplified using RT-PCR from the transfected mouse cells. Refining primer sequences enabled detection of mRNA covering the full *DUX4* ORF in differentiating myoblasts from FSHD patients but not unaffected controls [25], reinforcing the idea of *DUX4* expression as a potential pathogenic mechanism in FSHD.

## Detection of DUX4 protein

Characterization of DUX4 protein was difficult because the gene sequence was so GC rich that sequencing errors precluded clear definition of the ORF end. Initial studies of transcription/translation in vitro of a D4Z4 element indicated a 75-kDa protein in SDS-PAGE. This was predicted to be a dimer since the ORF sequence suggested a 42-kDa protein with 9 Cysteines potentially involved in dimerization [24]. Later, both the Rosa and Belayew/Coppée labs independently raised rabbit sera against synthetic peptides of the *DUX4* ORF that immunodetected a 52-kDa protein on a Western blot prepared with total protein extracts of cells transfected with constructs expressing *DUX4* or carrying 1 or 2 D4Z4 units [25]. However, the end of the DUX4 protein sequence could not be deduced from the ORF because of difficulties in its sequencing. The approach used by the Belayew/Coppée group was to get the DUX4 protein expressed by C2C12 cells transfected with the D4Z4 genomic fragments. Total cell proteins were separated on PAGE-SDS gels and those in the 50-kDa size range were extracted from the gel, digested with trypsin and the resulting peptides sequenced by MALDI-TOF to reveal the actual *DUX4* ORF; these data were added to Rosa’s publication on DUX4 toxicity [25].

The Belayew/Coppée group also developed the first mouse monoclonal anti-DUX4 antibody - MAb 9A12, raised against the 253 carboxyl-terminal residues. This protein domain was so toxic that bacteria had to be transformed with the inducible expression plasmid for each production. In combination with a new high sensitivity western blot immunodetection, MAb 9A12 identified a ~ 55-kDa DUX4 protein in primary FSHD but not healthy myotube cultures [26]. Unfortunately, this first DUX4 immunodetection only appeared as Supplemental Information (Fig. 2B). Since MAb 9A12 was the only antibody that could detect endogenous DUX4, the FSHD Society (USA) provided funding to produce and distribute it to ~ 20 research groups. MAb 9A12 was also used to validate new monoclonal antibodies developed by the Stephen J. Tapscott lab against different DUX4 domains [117]. However, because of its rare stochastic expression and rapid protein turnover [118], it is only recently that DUX4 could be detected in FSHD muscle biopsies by a highly sensitive proximity ligation assay with two monoclonal antibodies targeting different DUX4 domains [119]. Cytoplasmic DUX4 was also immunodetected in MRI-guided FSHD muscle biopsies with MAbs 9A12 and E5-5 in a cluster of activated satellite cells and in very few abnormal muscle fibres with features of abortive regeneration [120]. Although MAb 9A12 was raised against an antigen partly common to DUX4 and DUX4c, it does not detect endogenous DUX4c in muscle cells. Post translational modifications (PTM) may mask the DUX4c epitope but not affect the longer DUX4 protein, allowing DUX4 specificity [117].

Size differences on western blots for DUX4 proteins synthesized in vitro or in vivo result from specific PTM that can target 17 different residues in DUX4, as recently shown by Scott Harper’s group [121]. Of therapeutic interest, these researchers identified residues for which a change in PTM could suppress DUX4 toxicity: (i) increased phosphorylation of Ser/Thr residues by PKA or (ii) decreased Arg methylation by PRMT1.

## DUX4 RNA and protein are expressed in FSHD primary myogenic cultures

Using the optimized primers, the Belayew/Coppée group amplified the full length *DUX4* mRNA from total RNA of FSHD myotubes by RT-PCR. The cDNA sequence corresponded to *DUX4* transcription initiated in the distal D4Z4 unit and, unexpectedly, extended to the downstream pLAM region that provided a 3’ UTR with intron 2 and exon 3 with a PAS [26]. This PAS variant (ATTAAA) had not been previously detected by sequence analysis because it differed from the AATAAA consensus. Of note, a later analysis of multiple 3’ end sequencing data sets identified this variant in 14.5% of human mRNA 3’ ends, while the “consensus” PAS occurred in 47% [122]. Discovery of this PAS in pLAM in 2007 sparked discussion of whether *DUX4* mRNA transcribed from the most distal D4Z4 unit possessed a uniquely extended half-life for translation due to polyadenylation from this PAS, as well as how differences between 4qA and 4qB haplotypes could affect both splicing and polyadenylation [26].

Detection of *DUX4* mRNA in FSHD muscle cells had been so difficult up to this point that a reviewer requested a point to point comparison of conditions used for RNA extraction and RT-PCR alongside the methodology used by the 6 previous publications that could not detect *DUX4* transcripts [52, 94, 102, 107, 123, 124]. The optimized procedure detailed in Supplemental Table 3 [26] was used by the Tapscott group to independently confirm detection of a full length *DUX4* mRNA encoding DUX4-fl, and identify other spliced isoforms, including a shorter mRNA with the ORF limited to the homeoboxes encoding DUX4-s [112].

## DUX4 is a potent transcription factor

Misregulation of gene expression was observed in FSHD muscle, indicating possible perturbed regulation of transcription [125]. Yi Wen Chen performed RNA profiling of muscle biopsies from controls and 12 different neuromuscular disorders and found that *PITX1* RNA was specifically upregulated 11-fold in less affected, and 24-fold in clearly affected, muscles from FSHD patients [25]. PITX1 is a paired type homeodomain transcription factor involved in embryonic development and intriguingly, left/right asymmetry [126]. Although its expression level pointed to involvement in FSHD, *PITX1* maps to chromosome 5q31. As homeobox genes often act in regulatory networks the hypothesis was that DUX4 might activate *PITX1* expression. An evolutionarily conserved *cis*-element was identified in the murine *Pitx1* promoter, with a central TAAT core typical of homeodomain targets [26] and high similarity to the DUX1 binding site [87]. A mouse *Pitx1* promoter fragment carrying this homeodomain target sequence (but not a mutated version) bound DUX4 in vitro and activated expression of a linked reporter gene in C2C12 myoblasts, thus also creating the first DUX4 reporter gene [26]. Data from the Chen and Belayew/Coppée groups were pooled in a key publication showing *DUX4* mRNA extension to a PAS, detection of *DUX4* mRNA and protein (with MAb 9A12) in FSHD muscle extracts, and DUX4 function as a transcription factor with *Pitx1/PITX* proposed as the first DUX4 target gene [26]. These observations provided strong support for the relevance of DUX4 to FSHD pathology, with DUX4 and DUX4c metaphorically referred to as “pearls in the junk” [127].

Crucial evidence that *DUX4* was the culprit in FSHD was generated by Michael Kyba’s group who directly compared the effects of each of the then 6 proposed FSHD candidate genes (*FRG1*,* FRG2*,* TUBB4q*,* ANT1*,* DUX4* and *DUX4c*). Each was expressed individually from the same genetic locus in the iC2C12 myoblast model [128]. Strikingly, only DUX4 caused overt toxicity, with inhibition of the glutathione redox pathway and increased sensitivity to oxidative stress, as well as repression of the crucial muscle regulatory gene *MyoD*, MyoD target genes and myogenic differentiation [128]. Notably, pathways and processes perturbed by DUX4 over-expression were similar to those affected in FSHD myoblast cultures [102, 129], and altered expression of proteins linked to oxidative stress had also been found in FSHD muscle biopsies [130]. The DUX4 homeodomains had been noted to share a high degree of sequence similarity with paired box transcription factors [52], notably PAX3 and PAX7 [128]: master regulators of skeletal myogenesis [131]. Overexpression of Pax3 or Pax7 was shown to mitigate the ability of DUX4 to cause death in murine cells [128], leading to the idea that in FSHD, DUX4 also interferes with the capacity of PAX3 and PAX7 to regulate their target genes [128, 132].

Such engineered myoblast cell lines with inducible and variable transgenic *DUX4* expression proved an invaluable tool for DUX4 research, first in murine C2C12 myoblasts (iC2C12-DUX4) [128], and later in human LHCN-M2 myoblasts (LHCN-M2iDUX4) [133] and with a codon-optimised inducible *DUX4* transgene in human MB135 myoblasts (iDUX4) [134].

## A crucial role for the 4qA permissive allele in FSHD pathology

Nearly identical D4Z4 arrays on both chromosome 4 and 10 but only those on chromosome 4 being linked to FSHD, combined with the perceived ‘lack’ of detectable *DUX4* transcript, had been used to argue against a role for DUX4 in FSHD. In addition, the polymorphisms distal to D4Z4, with only the 4qA allele being associated with FSHD, remained intriguing, specifically because this sequence was > 98% identical to the homologous region in 10q26 [73, 74]. The answer however, rested on a single-nucleotide polymorphism (SNP) distal to the D4Z4 array. It had been found that *DUX4* mRNAs initiated from the most telomeric D4Z4 unit, extended to the flanking pLAM region that provided a non-canonical PAS to give *DUX4* mRNA a poly-A tail [26]. Later, a multicentre collaboration led by van der Maarel performed elegant genetic studies on a large population of patients with FSHD and unaffected relatives revealing that the ‘permissive’ 4qA allele carried this PAS, and that it was suppressed by a SNP in the non-permissive 4qB and 10qA alleles [23]. *DUX4* mRNA stability required polyadenylation, which allowed for DUX4 protein synthesis [23].

Further evidence of the 4qA permissive role subsequently emerged in the form of a family in which FSHD was attributed to a rearranged chromosome 10 containing a contracted D4Z4 array in which the non-permissive 10qA distal sequences had been replaced with a 4qA permissive region including the PAS. These genetic configurations led to *DUX4* expression from chromosome 10 causing FSHD [135]. Recently, human myoblasts were engineered to create the opposite sequence exchange i.e. replace the region distal to a contracted D4Z4 array on chromosome 4 with a non-permissive 10q distal region lacking the PAS, which improved the pathogenic cell phenotype [136].

## DUX4 to the fore by unifying FSHD1 and FSHD2 pathomechanisms

Requirement for both D4Z4 epigenetic derepression with DNA hypomethylation on contracted (FSHD1) and less contracted arrays (FSHD2) [76, 78] and a permissive 4qA haplotype supplying a PAS [23, 26] ‘unified’ FSHD1 and FSHD2 by a common pathomechanism: *DUX4* expression. This link was explained when the van der Maarel group demonstrated that FSHD2 was digenic [137]. In addition to an approximate 11-20 D4Z4 unit array on a 4qA allele, FSHD2 was frequently associated with mutations in *structural maintenance of chromosomes flexible hinge domain containing 1* (*SMCHD1*) on chromosome 18 [137, 138]. SMCHD1 is involved in epigenetic suppression of transcription of repeated elements and X-linked genes [139] and so SMCHD1 loss of function mutations favour DNA hypomethylation and open chromatin structure at D4Z4 and *DUX4 *transcription. *SMCHD1* also constitutes a disease modifier for FSHD1, some sequence variations explaining more severe clinical presentations than would be expected based on the patient’s number of D4Z4 units [140, 141]. Interestingly, Smchd1 favours DNA methylation by antagonizing ten-eleven translocation (TET) enzymes that initiate reversion of methylation, so Smchd1 suppression allows DNA hypomethylation, activation of *Dux* expression and establishment of a 2-cell like stage in mouse ES cells [142]. It was recently reported that SMCHD1 is required for activation of genes involved in myogenic differentiation and muscle regeneration, so SMCHD1 variations could also independently contribute to FSHD pathogenesis [143]. Furthermore, skeletal muscle may be more vulnerable to *DUX4* expression in general, as SMCHD1 protein levels dramatically decrease as myoblasts undergo myogenic differentiation [144].

The vast majority of FSHD2 cases are associated with mutations in *SMCHD1*, but other chromatin modifiers were found mutated in rare FSHD2 pedigrees, such as the DNA methyl transferase *DNMT3B* [145] and *LRIF1* [146]. Whole exome sequencing of clinically defined patients presenting either D4Z4 repeat array of typical short size or longer ones not usually associated to FSHD, recently identified further mutations in genes involved in chromatin structure that could contribute to epigenetic derepression at D4Z4, including *DNMT1*, *DNMT3A*,* EZH2*,* CTCF* and *SUV39H1* [147]. These genes may thus constitute disease modifiers for FSHD1 and FSHD2.

## Structural and functional domains of DUX4

The 424-residue DUX4 is actively transported into the nucleus [25, 148]. Harper and colleagues found that mutation of the first DUX4 homeodomain, suppressing DNA binding, prevented toxicity in zebrafish and mice [149]. Rosa’s group identified two nuclear localisation signals (NLS1 and NLS2), which along with the homeodomains and carboxyl-terminal domain, were also required for DUX4 cytotoxicity [108, 150]. Mutations affecting NLS1 and NLS2 and motifs IWF-65 in HD1 and IWF-140 in HD2 all decrease DUX4 cytotoxicity [150]. Given the similarity in sequence between the homeodomains in DUX4 and that in PAX3 or PAX7, the Kyba group reported that when DUX4 homeodomain HD1 was replaced by the mouse Pax7 homeodomain, DUX4 retained the ability to inhibit differentiation and induce cytotoxicity [151].

Chromatin immunoprecipitation combined with high throughput sequencing (ChIP-Seq) identified TAAYBBAATCA as the DUX4-binding site with two tandem homeodomain binding motifs (TAAT), separated by a single nucleotide [92]. Later studies showed that the optimal DNA sequence preferably bound by DUX4 is TAATCTAATCA, also being the most transcriptionally active sequence [152]. The crystal structure of tandem HD1 and HD2 bound to DNA revealed that they are arranged head-to-head. They also recognize different core sequences: HD1 binding TAAT (the HD1-altered target specificity unique to primates) and HD2 targeting TGAT [153]. Indeed, for transcription activity and cytotoxicity, only the two homeodomains and carboxyl-terminal region were required in a DUX4 ‘miniprotein’ [133].

The isolated carboxyl-terminal domain of DUX4 is a potent transcriptional activator [109], with most activity mapping to its last 20 residues [154]. DUX4 recruits the histone acetyltransferases p300/CBP to DUX4 target genes, allowing DUX4 to act as a ‘pioneer’ transcription factor, mediating a dramatic increase in acetylation at H3K27 and H3K18 to open chromatin at target genes [133, 155]. This powerful DUX4 transcriptional activity is also linked to interaction of a KIX motif in its carboxyl-terminal domain with a protein of the Mediator complex that could thus be recruited with RNA Polymerase II at DUX4 target promoters [156].

DUX4 can also indirectly regulate gene expression: it contains evolutionarily conserved LXXLL (NR box-like) motifs in its carboxyl-terminal domain [157, 158], which are also found in co-regulators of nuclear hormone receptors [158]. Rosa’s lab has demonstrated that DUX4 functions as a co-repressor of progesterone and glucocorticoid nuclear receptors [158], a phenomenon that may contribute to the sex differences observed in the onset and severity of FSHD [6]. Thus, in addition to its transcriptional activation role, the DUX4 carboxyl-terminal domain may also contribute to a potential endocrine function [158]. There is also a φXXφφ motif (AQPLL^388–392^) found in corepressors of hormone receptors (CoRNR boxes), which form a three-turn α-helical structure, similar to that observed in the predicted structure of the DUX4 carboxyl-terminal domain (Fig. 2C) [158].

In addition to its transcriptional functions in the nucleus, DUX4 protein unexpectedly interacts with cytoplasmic partners such as sarcomere Z-disk linked proteins desmin and LMCD1, as well as RNA-binding proteins C1QBP, SRSF9, RBM3, FUS/TLS and SFPQ [159, 160], which may contribute to its effects on RNA-processing [92, 161–163]. Many protein partners are shared by DUX4 and DUX4c, and so part of DUX4 toxicity could be linked to competition for partners normally associated with DUX4c [120]. DNA binding competition also occurs because of their identical homeodomains. DUX4c can compete for DUX4 target genes, for example those involved in the β-catenin pathway [164], but lacking a potent transcriptional activation domain, DUX4c would thus reduce DUX4 cytotoxicity [165].

Predictions of DUX4 3D structure in silico consistently show a largely disordered protein [154, 158], with α-helical regions in the two DNA-binding homeodomains (HD1 residues 19–78 and HD2 residues 94–153), and in the carboxyl-terminal region (residues ∼365–424), which includes the transactivation domain (Fig. 2C), missing in DUX4c.

In embryonic stem cells and mesenchymal stromal cells differentiating to osteoblasts or adipocytes [166], a *DUX4* mRNA was induced that initiated further upstream, encoding a 58-kDa protein from a 60-codon extended ORF starting at the initiator ATG proposed by Hewitt [52]. An additional 70-kDa DUX4 protein was also detected in cells but the ends of the encoding mRNA could not be mapped [24]. Neither of these DUX4 protein isoforms was toxic in the cell cultures used and their functions have not been reported [166].

## DUX4 transcriptional target genes and repetitive sequences

As a pioneer transcription factor, DUX4 has a cohort of target genes that could provide insight into its pathological functions. *PITX1* was the first DUX4 target gene identified [26]. Microarray transcriptional profiling on the murine iC2C12-DUX4 myoblast model to identify differentially expressed genes after 4 and 12 h of induction showed that most activated genes classified via gene ontology under ‘regulation of growth/development’ and ‘signal transduction’ [128].

To determine DUX4 target genes in human, the Tapscott group undertook microarray profiling of healthy human primary myoblasts transduced with a lentivirus expressing *DUX4*. Up-regulated genes were clearly involved with gamete/spermatogenesis, RNA polymerase II mediator complexes, and RNA splicing and processing, while down-regulated genes associated with immune response pathways [92]. Importantly, they identified several genes now considered to be canonical DUX4 target genes, including *ZSCAN4*,* PRAMEF1*,* TRIM43*,* MBD3L2* and *KHDC1* and showed up-regulation of these genes in FSHD muscle. DUX4 also bound and activated long terminal repeat (LTR) elements from a class of endogenous primate Mammalian apparent LTR-Retrotransposon (MaLR) and the related endogenous retroviruses (ERVs) family [92]. These retroviral DNA sequences, some of which have integrated near protein coding sequences, are normally epigenetically silenced except in early embryos where they can act as promoter/enhancers to express RNAs and proteins needed for early development. This pivotal paper also generated a useful DUX4 reporter using a 1.9-kb enhancer and promoter region of *ZSCAN4* that included four DUX4 binding sites, to drive luciferase expression [92]. The ‘newer’ technology of RNA-Seq was then used to measure transcription of DUX4 target genes [167, 168]. Because it binds to retrotransposon promoters, an issue in the study of *DUX4* expressed in mouse cells is that these differ between human and rodents, so that DUX4 also activates a unique set of genes in either species in addition to common targets [105, 169].

Why is it so difficult to detect DUX4 protein in FSHD muscle yet the consequences are so profound? DUX4 is difficult to detect because its expression is rare and random [161], estimated to be active in 1/1000 myoblasts [170] and 1/200 myonuclei in myotubes [118] at any one time. Skeletal muscle fibres are syncytial, which likely explains how such a low abundance protein causes a myopathy. *DUX4* transcripts produced in a few myonuclei are translated in the cytoplasm and the newly synthesized DUX4 proteins diffuse to adjacent nuclei, so activation of target genes is amplified [118].

## DUX4 activity as a molecular biomarker

A major bottleneck in FSHD research and drug development was the lack of sensitive and specific molecular biomarkers. Initially RNA expression profiling was performed on FSHD versus healthy muscle to define such specific biomarkers [171, 172]. While *DUX4* mRNA is detectable in muscle from FSHD foetuses [173, 174], it is much more difficult to detect in muscle biopsies from adult patients. Thus, indirect measures were developed to provide evidence that DUX4 is/was active in a muscle sample by quantifying mRNAs of activated DUX4 target genes as a DUX4 ‘footprint’ or ‘signature’. The first such footprint from Tapscott and colleagues consisted of 114 DUX4 target genes detected in FSHD but not control muscles [168], supporting the hypothesis that *DUX4* misexpression is a causal factor for FSHD. Christopher Banerji and Peter Zammit developed further signatures. DUX4 biomarkers include the “early” DUX4 signature from a study of human myoblasts overexpressing *DUX4* for 6 h from a doxycycline inducible promoter [133], consisting of 212 significantly upregulated transcripts [132]. The “late” DUX4 signature used a study of human myoblasts transduced with DUX4 lentivirus for 24 h [92], and comprises 165 significantly upregulated transcripts [132]. Given the similarity between the DUX4 and PAX7 homeodomains, a PAX7 signature was also derived from differential expression analysis consisting of 311 upregulated and 290 downregulated, PAX7 target genes [132]. Repression of the PAX7 target gene signature was found to be an equivalent biomarker for FSHD disease to activation of DUX4 target gene signatures in MRI-guided muscle biopsies [175]. Crucially, DUX4 target genes, the three validated DUX4 target gene signatures, MRI and histopathology measures failed to change in a cohort of FSHD patients with 1-year follow-up [176, 177]. In contrast, PAX7 signature repression increased over a year in paired FSHD samples, and so is a biomarker of FSHD progression over the relative short term, and so useful to monitor progress in clinical trials [176]. These observations also indicate that DUX4 interferes with PAX7 function in FSHD [4].

## Consequences of ectopic DUX4 activation

DUX4 activates so many target genes/repetitive elements that it is unsurprising that aberrant *DUX4* expression in muscle cells has many consequences, with the most obvious usually being rapid cell death. This was elegantly demonstrated by Dan Miller and colleagues, who used a DUX4-responsive nuclear GFP reporter gene in primary FSHD muscle cell cultures, where live imaging revealed rapid cell death after reporter gene activation by endogenous DUX4 [161]. Apoptotic pathways associated with DUX4-induced cell death were initially thought to be dependent on p53 [25, 149, 178] but this was later challenged [179, 180]. This discrepancy probably stems from the observation that p53 can activate *DUX4* expression by interaction with an enhancer in a LTR 18 kb 3’ of the *DUX4* gene in FSHD iPSC cells [83].

*DUX4* expression in human myogenic cells also correlates with the accumulation of DNA damage [181, 182], and DUX4-expressing cells exhibit impaired DNA damage response [183]. DUX4-induced cellular toxicity is linked to accumulation of double stranded transcripts of human satellite II DNA (HSATII) [167] and induces formation of intranuclear HSATII dsRNA foci that bind and sequester nuclear proteins [180]. Interestingly, DUX4 and HSATII expression are highly correlated during early human embryonic development [182], suggesting a normal role for DUX4 in these mechanisms.

Reactive oxygen species (ROS) were shown to induce *DUX4* gene expression [184] and earlier studies described known signs of oxidative stress damage in FSHD muscle cells [102, 129, 130, 185]. DUX4 increases sensitivity to oxidative stress by repression of the glutathione redox pathway [128] and disruption of mitochondrial function [130, 186]. FSHD muscle biopsies or primary myoblast cultures show altered expression of proteins linked to oxidative stress [102, 129], not only because of DUX4 but also possibly due to ANT1 misexpression [187]. ROS cause DNA breaks, which activate p53, leading to the DNA damage response (DDR). These findings combined, suggest that p53 is not only induced by DUX4/DUX4 target genes but may also directly trigger *DUX4* expression in some cells or stages of development, potentially indicating a vicious cycle of ROS-p53-DUX4-ROS. Addition of antioxidants to DUX4-transfected, and FSHD, myoblasts reduced both DNA damage and morphological defects in myotube formation, suggesting that these pathological phenotypes are due to oxidative stress [181, 188]. Intriguingly *PITX1* is among the DUX4-activated genes decreased by antioxidants, suggesting a role of NRF2, the transcription factor involved in cyto protection against oxidative stress [188]. A study on DUX4 binding had indicated that the homeodomain binding site conserved in the *PITX1/Pitx1* gene is not optimal [152], so maybe combination of weak DUX4 and NRF2 activation explains the strong PITX1 increase previously observed [26].

FSHD myoblasts also show defective myogenic differentiation into myotubes [102], and primary FSHD myotubes were described as often having either an ‘atrophic’ or ‘disorganised’ morphology [189]. Indeed, ectopic *DUX4* expression inhibits differentiation and causes such ‘atrophic’ myotubes [178] although a later time lapse study revealed that FSHD myotubes were actually hypotrophic [190]. This is likely related to the observation that in mouse, DUX4 downregulates *MyoD* and its target genes [128], creating a more stem-cell like transcriptome [105] and in human cells, DUX4 inhibits *MYOD* and *MYF5*, despite binding to the *MYF5* enhancer [191].

Numerous other cellular activities are also perturbed by DUX4, including RNA metabolism/processing and translation [92, 161, 163] and immune responses [92], indicating that DUX4 initiates a cascade of dysregulated gene expression with multiple and interconnected processes affected [192, 193].

## Modelling DUX4 function in vivo

A major limitation of animal models to investigate DUX4 function in vivo is that the D4Z4 tandem repeats and *DUX4* are only strongly conserved in old world primates [111, 194] and so there is no ‘natural’ equivalent in most standard model animals [195]. Thus, the genetic and epigenetic mechanisms underlying the rare, stochastic expression of *DUX4* in FSHD are difficult to recapitulate in vivo [196]. There is also the issue of the limited overlap between DUX4 target genes in human and standard models such as the mouse [105, 169], and the fact that many repetitive elements and other retrotransposons are only found in human [92]. Finally, DUX4 is highly toxic to most cells in most organisms [25].

### Non-mammalian DUX4 animal models

Ubiquitous *DUX4* expression was found incompatible with normal Drosophila, zebrafish and Xenopus development, thus limiting their use [112–114]. The Harper lab showed that muscle-directed transgenic *DUX4* expression produced viable zebrafish with approximately half of the embryos malformed and with defective muscle structure [149]. Injecting low levels of *DUX4* mRNA at the zebrafish one cell stage resulted in asymmetric muscle disorganisation and degeneration. Interestingly, the homeodomain-containing *DUX4-s* mRNA reduced toxicity of DUX4-fl [197]. However, recombination-controlled muscle specific *DUX4-mCherry* expression in zebrafish enables onset of DUX4 to be controlled and visualized [198].

### Mammalian DUX4 animal models

After much effort and frustration due to cryptic Sp1-dependent promoters in GC rich sequences allowing for *DUX4* expression, DUX4 toxicity and the normal limited expression window in early embryogenesis [199], murine DUX4 models finally emerged. Harper and colleagues used intramuscular injection of AAV-*DUX4*, leading to local myofiber degeneration, infiltrating mononuclear cells, and p53-dependent apoptosis [149]. The van der Maarel group created transgenic mice using the lambda-42 phage containing an FSHD patient-derived genomic fragment with 2.5 repeats of *D4Z4* and the PAS associated with the permissive 4qA haplotype [196]. Their single D4Z4-2.5 mouse line had relaxed chromatin and hypomethylation with *DUX4* mRNA in testes, embryonic cells and skeletal muscle tissues. No alterations in muscle structure or function were found in D4Z4-2.5 mice [196] but *DUX4* expression was up-regulated during muscle regeneration [105].

The Kyba lab generated the iDUX4 [2.7] mouse line with ubiquitous doxycycline inducible (rtTA driver) *DUX4* expression. However, the promoter was leaky, and use of a SV40 PAS linked to the *DUX4* gene led to very efficient mRNA polyadenylation and stability, leading to high DUX4 protein levels and lethality. Rare surviving males had smaller and weaker, but not overtly dystrophic, muscles and a skin phenotype, before dying after ~ 2 months [200, 201]. Instead, the iDUX4pA mouse had a doxycycline-inducible genomic fragment from an FSHD 4qA161 allele that included the terminal D4Z4 repeat with the *DUX4* ORF and 3ʹ UTR with the less effective native PAS, inserted into the X chromosome [202]. iDUX4pA mice had skin hyperkeratosis, alopecia and high-frequency hearing impairment. Males were less active, with atrophic, weaker muscles with extremely low levels of *DUX4* mRNA and some DUX4 target gene expression, living only ~ 4 months. However, mice died after doxycycline induction necessitating use of a muscle-specific HSA rtTA driver to create the iDUX4pA-HSA strain [202]. High doxycycline doses trigger severe muscle damage with loss of ambulation, while low level chronic induction causes progressive muscle atrophy and weakness, with hallmarks of FSHD histopathology. Differential gene expression profiles of iDUX4pA-HSA mice and FSHD muscle have significant overlap [202, 203].

Mouse models quickly followed in which *DUX4* expression was activated following recombination. Harper and colleagues developed the TIC-DUX4 mice that employed a DNA fragment encoding V5-tagged DUX4 and the natural *DUX4* 3′ UTR [204]. The Jones lab produced the FLExDUX4 mouse, with a modified *DUX4* transgene encoding only DUX4-fl with both native 5′ and 3′ UTRs [205]. Both constructs were inserted into the *Rosa26* locus and mice crossed to mice with muscle-specific *Cre* expression [206]. Older TIC-DUX4 mice exhibit low-level transgene expression but on exposure to tamoxifen, mice develop an FSHD-like muscle pathology. FLExDUX4 mice have mild muscle-wasting phenotype but recombination causes muscle pathology with FSHD characteristics. As with the iDUX4pA-HAS mice, *DUX4* expression is conditional and titratable. Varying tamoxifen dose allows for control over time of onset and severity of muscle phenotypes. A simple non-transgenic mouse model was also developed with local intra-muscular injection/electroporation of naked plasmid DNA expressing *DUX4* into the *tibialis anterior* that causes well delineated muscle lesions after a week [207].

Jones and collaborators have generated a large animal model using the *ROSA* locus to drive *DUX4* expression after Cre-mediated recombination in Gottingen minipigs [208]. A pig model also has the advantage that both porcine DUXC and human DUX4 activate a very similar early embryonic program in porcine myogenic cells [209]. However, in all these inducible mammalian models, the underlying regulation of *DUX4* expression is not by the native human locus.

### Xenograft models of FSHD and DUX4

There is debate about how closely these non-primate inducible DUX4 models generating a ‘DUX4-opathy’ reproduce FSHD pathology [4]. Xenograft models allow examination of human *DUX4* expressed from its native promoter and surrounding 4q35 DNA sequences in vivo. FSHD muscle tissue grafted into mouse generated various proportions of human cells in hybrid regenerated mouse muscle fibres [210]. Instead, xenografting immortalized FSHD muscle precursor cells into mouse resulted in organized and innervated human muscle fibres (with minimal contribution of murine myonuclei) and expression of *DUX4* and DUX4 target genes. Satellite cells were also reported to be present [211]. An issue with these xenograft models though, is variability in the amount of FSHD muscle made in each graft.

## DUX4 contributes to zygote genome activation

An outstanding question was why does DUX4 have such pleiotropic effects? Another enigma was why the *DUX4* coding region has been conserved in “junk” repeat DNA through evolution for over 100 million years [111], which hinted at a functional role. This was uncovered in 2017 in back-to-back publications from the Bradley Cairns, Stephen J. Tapscott and Didier Trono groups who reported that DUX4 was involved in human zygote genome activation (ZGA): waves of embryonic gene transcription during early embryogenesis. The DUX4 functional homolog *Dux* plays a similar role in mouse [212–214]. During the cleavage stage following fertilisation, the human zygote undergoes cell divisions that are regulated by maternal RNAs and proteins from the oocyte. DUX4 accumulates during this period, peaking at the 4-cell stage in human and participates in the minor wave of ZGA, activating genes required for both trophectoderm and embryonic development, contributing to establishment of totipotent cells. As a pioneer transcription factor, DUX4 was found to induce genes transiently expressed during the cleavage stage, including *ZSCAN4*, *KDM4E* and *PRAMEF*, many of which are transcribed when *DUX4* is expressed in myogenic cells [92]. DUX4 also activates expression of repetitive elements, including pericentromeric HSATII repeats, MaLRs, ERVs, and long interspersed nuclear elements (LINE1). Binding to such repetitive elements, DUX4 sometimes creates unorthodox promoters and first exons for nearby genes, or novel transcription start sites for long non-coding RNAs or antisense transcripts [167, 180, 182]. After its activity in the early embryo, *DUX4* is epigenetically silenced, which remains throughout life, although *DUX4* transcript and protein are detectable in some human tissues with high levels of apoptosis such as testis and thymus [170, 215]. *DUX4* is also expressed in late differentiating keratinocytes [216], mesenchymal stem cells from umbilical cord and adipose tissue differentiating to osteoblasts [166]. However, there is no absolute requirement for a *Dux* gene in mice [217, 218] since redundancy with homeodomain protein Obox4 allows development of *Dux*-null embryos [219].

In mouse, rRNA synthesis and nucleolar maturation at the 2-cell  stage limits the window of *Dux* activity [199], and Smchd1 binds the *Dux* gene to contribute to epigenetic repression [142, 220]. RNA of the LINE1 retrotransposon can act as a nuclear scaffold to recruit Nucleolin and Kap1 to facilitate *Dux* silencing for both ribosomal RNA gene transcription and exit from the 2-cell stage [221]. Such mechanisms may also be employed to control DUX4 at the 4–8 cell stage in human. In addition, a Dux inhibition loop is mediated by DuxBL a rodent homologue lacking an activation domain that is induced by Dux at the ZGA and then silences Dux-induced genes, allowing development to progress [222]. Again, a similar system may occur in human embryos with DUXA, a truncated DUX4 homologue [223], although other researchers propose that DUXA can activate DUX4 target genes in FSHD2 late muscle cell differentiation [224].

These observations also highlighted that there is a PAS that can be employed even with a 4qB haplotype, which was proposed to be in exon 7 [170]. The embryonic transcriptional program activated by the potent DUX4 pioneer transcription factor likely explains the many disparate effects described in FSHD muscle including interference with metabolism, RNA processing and myogenic differentiation.

## Other pathological roles of DUX4

The novel chromosomal translocation generating hybrid CIC–DUX4 proteins in Ewing-like sarcomas signalled a potentially wider role for DUX4 in pathology [109]. Later, chimeric transcription factors involving DUX4 were found in another cancer, a subset of B cell acute lymphoblastic leukaemia (B-ALL) [225–228]. Some chromosomal rearrangements in B-ALL were characterised by insertion of D4Z4 repeats into the *IGH* locus. This encoded a hybrid transcription factor termed DUX-IGH incorporating the DUX4 amino-terminal region with the two homeodomains that dictate target genes selection, and a unique carboxyl-terminus [225]. It is of note that this chromosomal rearrangement also contains regulatory elements associated with the D4Z4 units. Expression of *DUX-IGH* in B-cells/their precursors in B-ALL is consistent with observations that DUX4 and transcripts of its target genes can also be detected in immortalised B-cell lymphoblastoid clones from FSHD patients, although their immortalisation with Epstein–Barr virus is a confounding factor [229, 230], and *DUX4* expression was not detected in primary FSHD peripheral blood cells [231].

Crucially, *DUX4* is reactivated in some solid cancers via *cis*-acting inherited genetic variation and *trans*-acting somatically acquired mutations in repressors. DUX4 induces a metastable early embryogenic stem cell transcription (ZGA, 8 cell-like program, markers of early embryogenic lineages) [232] and causes immune evasion of the cancer cells by reducing antigen presentation, since it prevents IFN-γ-mediated induction of MHC class I genes [233]. This occurs via the LXXLL (NR box-like) motifs in the carboxyl-terminal domain of DUX4 interacting with STAT1 to suppress IFNγ-induced genes by reducing bound STAT1 and RNA Pol-II [234]. *DUX4* is expressed in ~ 10–50% of advanced bladder, breast, kidney, prostate, and skin cancers, revealing its high frequency in metastatic tumours, and is associated with shorter survival times [235].

Finally, *DUX4* expression is induced by Herpes viruses, thus mimicking an early embryonic-like transcriptional program that prevents epigenetic silencing of the viral genome and facilitates viral gene expression and viral proliferation [236, 237].

Interestingly, *DUX4* mRNA was only identified as deregulated by transcriptome analysis in other biological systems than FSHD muscle after its gene was considered functional [25, 26], and its sequence was excluded from the “gene-less repetitive regions” (“junk DNA”) not considered by the Repeat Masker software.

## DUX4 today

Much focus is now on understanding the regulation of DUX4, its effects in healthy and FSHD cells, and targeting DUX4 therapeutically. A few clinical trials had been performed in patients with FSHD to evaluate various therapeutics used in other neuromuscular disorders. However, when DUX4 became widely suspected as the main cause of FSHD, focus shifted to strategies to prevent/reduce DUX4 effects by disrupting *DUX4* gene expression, blocking translation of *DUX4* transcripts, and/or interfering with protein function [238].

However, many questions remain about DUX4 function in health and FSHD, including:

- If the mean prevalence of FSHD is 5/100,000 [1], yet approximately 13/1000 healthy individuals carry alleles with 4–8 D4Z4 repeats with a 4qA haplotype [62], then why are only 1/260 people with a ‘pathogenic allele’ affected by FSHD?
- What is the normal physiological role of DUX4 in spermatocyte precursors, keratinocytes and thymic cells etc?
- Does DUX4 play a normal role in regulating hormone receptors, and does it disrupt the endocrine physiology of muscle? What is its interplay with estrogens that were shown to inhibit its toxicity in cell cultures [239]?
- What is the importance of DUX4 in non-myogenic cell types such as FAPs, macrophages, lymphocytes in FSHD pathology? What are the mechanisms by which DUX4 affects immune response and contributes to inflammation?
- What are the functions of DUX4, DUX4c and other DUX proteins in rRNA synthesis, processing and ribosome assembly at the nucleolus?
- Does DUX4 participate in DNA damage and/or normal DNA repair processes related to the occurrence of double strand breaks?
- Why are there larger DUX4 isoforms found in ESCs and MSCs? Why are these not cytotoxic?
- Can DUX4 diffuse between cells? Several homeoproteins can cross the plasma membrane for paracrine activity [240].
- Are DUX4 and DUX4c expressed simultaneously in a given nucleus and what are the consequences?
- Where, when and how does DUX4 interact with PAX7?
- Will therapeutic suppression of DUX4 in adult patients with FSHD slow or suppress pathology and allow muscle regeneration?
- Will targeting DUX4 in FSHD have deleterious effects on specific cell types in testis, skin and thymus?

## Summary

**Fig. 3:**
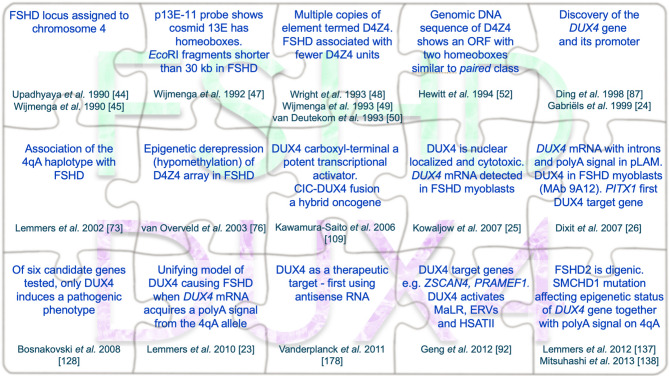
The Puzzle that is the involvement of DUX4 in FSHD Schematic showing some of the evidence that contributed to completing the jigsaw that was the identification of the involvement of DUX4 in FSHD pathology

Here, we have detailed the emergence of DUX4 from “junk DNA” status to its role in FSHD pathology (Fig. 3). Contraction of the D4Z4 array and/or mutation in epigenetic modifiers triggers re-expression of the *DUX4* retrogene, whose RNA is stabilised by addition of a poly-A tail because of the PAS present on a 4qA haplotype. In skeletal muscle, this early embryonic transcription factor reactivates a totipotent stem cell program that perturbs the tissue-specific gene expression profile to ultimately cause muscle damage, weakness and wasting.

Many researchers participated in the initial findings that chromosome 4 harbours the FSHD disease locus and identifying the microsatellite array of D4Z4 units near the 4q telomere and the *DUX4* ORF. This was followed by the discovery that epigenetic derepression and *DUX4* expression from the distal unit was the culprit. As the mechanisms underlying the complex FSHD pathogenesis unfolded, much work then went into proving *DUX4* was expressed in FSHD and defining its functions (Fig. 3). DUX4 is now not only central to FSHD research but roles in early embryogenesis, cancer and viral infection are widening interest in this enigmatic transcription factor. The steady increase in publications and citations since the discovery of the *DUX4* gene in 1999 [24] testifies to the prominence DUX4 has gained. The total from 1999 to 2024 is now 783 publications and 22,254 citations (publications with ‘DUX4’ in an ‘All fields’ search via ‘Web of Science’ accessed on 31/12/2024). As with the last quarter of a century, the next 25 years of DUX4 research will be equally fascinating!

## Data Availability

No datasets were generated or analysed during the current study.
